# Present Aspect of Medical Science

**Published:** 1870-07

**Authors:** N. J. Davis


PRESENT ASPECT OF MEDICAL SCIENCE.—By N. J. Davis.—The field of medical knowledge may be divided into two departments. The first embraces all that relates to the causes, nature, tendencies and results of disease; and the second all that relates to the nature and application of remedies. Disease being simple deviations from the natural healthy condition of some function or structure belonging to the human system, it is obvious that the first step in their study is to acquire a thorough knowledge of the healthy condition and function of every organ and structure that helps to work up our organization. To accomplish this the dissecting knife of the anatomist must be wielded with sufficient patience and skill to trace the existence, boundaries and connections of every bone, ligament, muscle, nerve, blood vessel, membrane, etc., that exists in the human body. Each anatomical structure thus developed with the dissecting knife, must be still further studied, and the special arrangement of its atoms or cells, too minute for observation with the eye, must be revealed under the magnifying power of the microscope. Finally, to complete the object of our study, by ascertaining the elementary substances that enter into the composition of each tissue, we must call into requisition the crucible and test glass of the chemist.—*Indiana Journal of Medicine.*

---

THE question of the origin of the white corpuscles of the blood is one to which it is by no means easy to give a satisfactory reply. A communication, however, has lately been made by Dr. Klein to Virchow's *Archiv*, which goes far to show that they result from the fission of pre-existing corpuscles.—*London Lancet.*

